# Midlife Risk Factors for Impaired Physical and Cognitive Functioning at Older Ages: A Cohort Study

**DOI:** 10.1093/gerona/glw092

**Published:** 2016-06-06

**Authors:** Eric J. Brunner, Catherine A. Welch, Martin J. Shipley, Sara Ahmadi-Abhari, Archana Singh-Manoux, Mika Kivimäki

**Affiliations:** ^1^Research Department of Epidemiology and Public Health, University College London, UK.; ^2^INSERM U1018, Center for Research in Epidemiology and Population Health, Paul Brousse Hospital, Villejuif, France.

**Keywords:** Physical functioning, Cognitive functioning, Life course, Aging

## Abstract

**Background:**

Previous studies examined midlife risk factors separately for old-age impaired physical and cognitive functioning. We determined the overlap of risk factors for both domains of functioning within the same setting.

**Methods:**

Biological and behavioral risk factors at age 50 years and cognitive and physical functioning were assessed 18 (*SD =* 5) years later in the Whitehall II study (*N* = 6,316). Impaired physical functioning was defined as ≥1 limitation on the activities of daily living scale. Impaired cognitive functioning was defined as Mini-Mental State Examination score <27. Two statistical analyses were employed: minimally adjusted analysis (for age, sex, and ethnicity) and mutually adjusted analysis (including all risk factors). Missing data on risk factors were imputed.

**Results:**

After confounder adjustment, impaired physical and cognitive functioning at older ages were predicted by hypertension (odds ratios [ORs] 1.80 95% confidence interval [CI] 1.39–2.33 and 1.57 95% CI 1.07–2.31, respectively), poor lung function (1.51 95% CI 1.28–1.78 and 1.31 95% CI 1.08–1.59), and physical inactivity, marginally in the case of cognitive function (1.50 95% CI 1.19–1.90 and 1.27 95% CI 0.99–1.62) at age 50 years. Impaired physical functioning but not cognitive functioning was additionally predicted by depression and higher body mass index (1.72 95% CI 1.46–2.03 and 1.29 95% CI 1.16–1.44, respectively).

**Conclusions:**

Several midlife risk factors are associated with impaired physical and cognitive functioning in old age, supporting a unified prevention policy. Analysis of 12 risk factors at age 50 suggests that strategies targeting physical inactivity, hypertension, and poor lung function will reduce impairments in both cognitive and physical functioning in old age.

An important policy goal in respect of healthy aging is to increase disability-free life expectancy and compress age-related morbidity into a short period before death (1). Several modifiable risk factors, including midlife raised blood pressure (2) and physical inactivity (3), have been identified for clinical management to reduce or delay impaired physical or cognitive functioning, but few studies have examined the extent to which such factors affect both physical and cognitive domains of functioning.

To inform research, the World Health Organization proposed the International Classification of Functioning, Disability and Health (ICF) (4), which synthesizes the medical and social conceptual models of disability and combines the individual and environmental dimensions of disablement within a unified biopsychosocial framework. Broadly, impairment arises when physiological or cognitive problems occur, resulting in activity limitation. Previous studies have examined risk factors either for impaired physical functioning or for impaired cognitive functioning (5–8), but did not identify predictors of both types of functional loss. Consequently, the associations of several putative risk factors which affect these two domains of functioning remain unclear.

To address this gap in the evidence, associations of 12 modifiable behavioral and biomedical risk factors at age 50 years with future impaired physical and cognitive functioning were assessed 18 (*SD =* 5) years later among participants in the Whitehall II study.

## Methods

### Data Source

We analyzed data from the Whitehall II study (9). The study started in 1985 and included 10,308 civil servants aged 35–55 years, with repeated data collection every 2–3 years. Participants completed a health and lifestyle questionnaire at each data collection and underwent a clinical screening at alternate assessments.

### Outcome Definitions

We defined impaired physical functioning according to the activities of daily living (ADL) scale (10), measuring difficulties with six self-care tasks: dressing, walking across the room, bathing or showering, eating, getting in or out of bed, and using the toilet. The ADL scale corresponds to the ICF model activity domain (Supplementary Figure 1). We defined impaired physical functioning as at least one limitation on the ADL scale. We defined impaired cognitive function using the Mini-Mental State Examination (MMSE) (11). The MMSE belongs to the ICF model body function and structure domain. The range of MMSE scale is 0–30. We dichotomized MMSE to indicate impaired cognitive functioning if MMSE score is less than 27 (12).

We identified the first occurrence for impaired physical and cognitive functioning separately at either the fifth (2007–2009) or the sixth clinic (2012–2013). We investigated the outcome definitions by plotting the distribution of both outcome measures by sex.

### Risk Factors

We extracted risk factors recorded during data collection when participants were aged 50 years, or the closest collection within ±5 years. If age 50 years was exactly in between two data collections, we selected the one that included a clinical screening.

Nonmodifiable risk factors, from the ICF personal factors domain (Supplementary Figure 1), were as follows: age at impaired physical or cognitive functioning, time from age 50 to impaired physical or cognitive functioning, sex, ethnicity, marital status at age 50 years, highest ever academic qualification, and last known employment grade (equivalent to civil service grade if no longer employed at civil service).

Modifiable behavioral risk factors at age 50 years, from the ICF personal factors domain, were as follows: daily fruit and vegetable consumption (1+ portions/day), smoking status (current, ex, or never smoker), alcohol consumption (none, moderate [women 1–14, men 1–21 units/week], or heavy [women ≥ 15, men ≥22 units/week] consumption), and average hours of sleep on a weeknight dichotomized as normal (7–8 average hours on a weeknight) or abnormal. Physical activity (13) was categorized as “sufficiently active” (≥2.5 hours/week moderate or ≥1 hour/week vigorous physical activity), “inactivity” (<1 hour/week moderate and <1 hour/week vigorous physical activity), or “moderately active” (if not active or inactive).

From the ICF health condition domain at age 50 years, we identified externally verified stroke and myocardial infarction, and depression if the 30-item General Health Questionnaire identified depressive symptoms (14).

We investigated modifiable biomedical risk factors at age 50 years from the body function and structure domain. The following risk factors were derived from measures recorded during the clinic: body mass index (BMI [kg m^−2^]), also dichotomized to create an obese group (BMI ≥ 30kg m^−2^), hypertension (systolic blood pressure ≥ 140 mmHg, diastolic blood pressure ≥ 90 mmHg, or reporting antihypertensive drug treatment), total:high-density lipoprotein cholesterol ratio, fasting glucose (mmol L^−1^), forced expiratory volume in 1 second (FEV_1_, L), and C-reactive protein (CRP) level (mg L^−1^). FEV_1_ was first measured at the fourth clinic (2002–2004) based on standardized methods (15). We used the largest FEV_1_ value from three maneuvers. Lung volumes are related to body size, and standing height is the most important correlating variable, so we corrected FEV_1_ for height by dividing by the square of the participant’s standing height and multiplying by the square of the sample mean height, 1.77 m in men and 1.63 m in women (16), to ensure observed variation in lung function was due to factors other than body size.

### Statistical Analysis

The study sample was defined as those who participated at the fifth (2007–2009) or sixth (2012–2013) clinic and who provided ADL and MMSE data. We summarized risk factors at age 50 years by outcome category. We used χ^2^ tests to test for heterogeneity of prevalence in each exposure group. We imputed missing risk factor data using multiple imputation which replaces missing values with randomly selected draws from the missing data distribution conditional on the observed data, specified by an imputation model, creating multiple imputed data sets (17). Multiple imputation accounts for uncertainty due to missing data and obtains unbiased estimates and standard errors if the missing at random assumption is plausible, that is, the reason for the missing data is associated with observed, but not with unobserved, data (17). The imputation model included the risk factors described earlier, impaired physical and cognitive functioning outcomes, and the following auxiliary variables measured at age 50 years, associated with at least one risk factor with missing data (18): data wave when participant was aged 50 years, angina or chest pain, antihypertensive drugs, mild exercise, satisfaction with standard of living, employment status, family history of high blood pressure, angina, diabetes, myocardial infarction or stroke, diastolic blood pressure, waist circumference, forced vital capacity, and diabetes status. Additionally, for repeatedly measured variables, we included observed measurements from adjacent data collections to age 50 years. We imputed missing values with 10 cycles to generate 20 imputed data sets, analyzed separately, and combined the results using Rubin’s rules (17).

We fitted logistic regression models for impaired physical and cognitive functioning, respectively, to estimate the associations between each risk factor at age 50 years and each binary old-age functioning outcome. We initially adjusted for age, sex, and ethnicity and then mutually adjusted for all modifiable and nonmodifiable risk factors at age 50 years. BMI and obesity (BMI ≥ 30) were fitted in separate alternative models. We used Cochran’s *Q* test to examine heterogeneity of coefficients. As a sensitivity analysis, we compared the analysis of multiply imputed data with the last observation carried forward (LOCF) method: replacing missing values with the closest recorded value before age 50 (or if missing, after age 50), excluding participants who still had missing values. We assumed participants without ethnicity data were White. Analyses were performed using Stata 13.1.

## Results

Of the original cohort, 9,354 were alive at the fifth clinic, of whom 7,018 (70.9%) participated at the fifth or sixth clinic. We excluded 638 participants with missing physical or cognitive functioning data and 64 participants lacking baseline data at age 50±5 years. The analytic sample consisted of 6,316 individuals (90.0% of participants): 980 (15.5%) with impaired physical functioning and 927 (14.7%) with impaired cognitive functioning (MMSE < 27). Of these, 97 (10.5%) had an MMSE score of less than 24. The distribution of both outcome measures showed that a reasonable number of participants had impaired physical functioning and impaired cognitive functioning using these definitions (Supplementary Figure 2). We showed distributions of impaired physical and cognitive functioning for the whole cohort as these distributions were similar for male and female participants. ADL data were not available at baseline because the questionnaire was added to the protocol when participants were aged 55 years or older. Among the small number of participants in the baseline data who completed the MMSE, few scored less than 27 (*n* = 2/75, 2.7%). Length of follow-up for physical and cognitive functioning was mean 18.3 (*SD =* 5.2) and 18.6 (*SD =* 5.1) years, respectively, with minimum follow-up time of 4.9 years.

Demographic and socioeconomic (nonmodifiable) risk factors were associated with impaired physical and cognitive functioning (Table 1). Participants in higher civil service grades were higher functioning compared with their lower grade counterparts. All nonmodifiable risk factors at age 50 were significantly associated with both impaired physical and cognitive functioning, except ethnicity, marital status, and highest academic qualification.

**Table 1.** Prevalence of Impaired Physical and Cognitive Functioning at Time of Outcome Measurement (2007–2009 or 2012–2013) by Nonmodifiable Participant Characteristics (with row percentages)

	Total (*N* = 6,316)	Impaired Physical Functioning (*n* = 980)		Impaired Cognitive Functioning (*n* = 927)	
		*n* (%)	*p* Value^a^	*n* (%)	*p* Value^a^
Age group (years) at time of outcome measurement					
55–64	1,795/1,802^b^	277 (15.4)	<.001	175 (9.7)	<.001
65–69	1,834/1,855	231 (12.6)		213 (11.5)	
70–74	1,375/1,390	242 (17.6)		271 (19.5)	
75–79	1,092/1,042	194 (17.8)		229 (22.0)	
80+	220/227	36 (16.4)		39 (17.2)	
Sex					
Male	4,516	653 (14.5)	<.001	603 (13.4)	<.001
Female	1,800	327 (18.2)		324 (18.0)	
Ethnicity^c^					
White	5,809	895 (15.4)	.655	701 (12.1)	<.001
South Asian	292	50 (17.1)		129 (44.2)	
Black	160	29 (18.1)		84 (52.5)	
Missing	10	0 (0.0)		2 (20.0)	
Marital status at age 50 years					
Married/cohabiting	4,736	676 (14.3)	<.001	686 (14.5)	.207
Single	752	144 (19.1)		114 (15.2)	
Divorced/widowed	512	108 (21.1)		89 (17.4)	
Missing	316	52 (16.5)		38 (12.0)	
Highest ever academic qualification					
Degree	2,520	336 (13.3)	<.001	280 (11.1)	<.001
School	3,052	494 (16.2)		462 (15.1)	
None	463	100 (21.6)		130 (28.1)	
Missing	281	50 (17.8)		55 (19.6)	
Last known grade					
1–2 (High)	2,717	359 (13.2)	<.001	217 (8.0)	<.001
3–5	2,798	447 (16.0)		418 (14.9)	
6 (Low)	801	174 (21.7)		292 (36.5)	

*Notes:* Impaired physical functioning—at least one activity of daily living. Impaired cognitive functioning—Mini-Mental State Examination score <27. Some participants have both impaired physical and cognitive functioning.

^a^χ^2^ test for heterogeneity of prevalence in each exposure group.

^b^The figures in the age group total column are totals for impaired physical functioning and impaired cognitive functioning respectively.

^c^Forty-five participants with “other” ethnicity.

Several health behaviors and biomedical risk factors at age 50 years were associated with impaired physical and cognitive functioning (Supplementary Table 1). Physical activity, current smoking, low fruit and vegetable consumption, hypertension, and lower FEV_1_ were each associated with both types of functional impairment.

In minimally adjusted models, we included age, sex, and ethnicity. Impaired physical and cognitive functioning at follow-up were predicted by physical inactivity, hypertension, and lower FEV_1_ at age 50 years, for example, physical inactivity predicted impaired cognitive function, odds ratio (OR) 1.52 95% confidence interval (CI) 1.21–1.91 (Supplementary Tables 2 and 3 and Supplementary Figure 3). There was no evidence of heterogeneity in these risk factor effects. Impaired physical functioning, but not impaired cognitive functioning, was predicted by fruit and vegetable consumption, current smoking, sleep duration, depression, obesity/higher BMI, total:high-density lipoprotein cholesterol ratio, fasting glucose, and serum CRP at age 50 years, with heterogeneity of risk factor effects being evident for depression, obesity/higher BMI, fasting glucose, and serum CRP.

**Figure 1.**
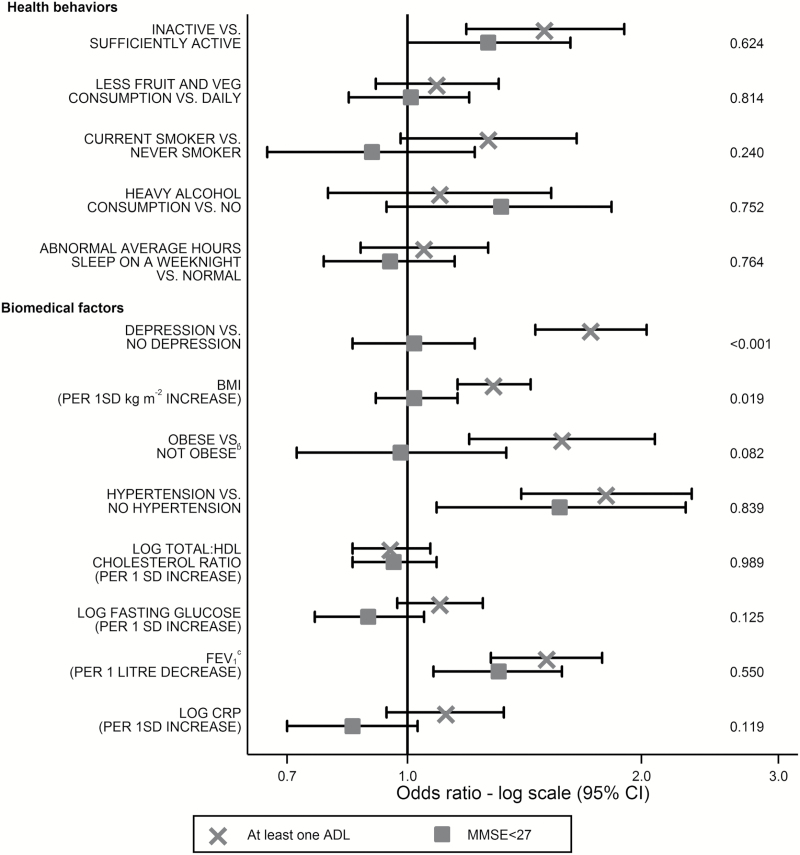
Prospective associations of modifiable risk factors at age 50 years with impaired physical and cognitive functioning in 2007–2009 or 2012–2013. *N* = 6,316. Model with mutual adjustment for all risk factors shown in the figure^a^, plus age, sex, ethnicity, education, marital status, and last known grade at age 50 years. ADL = activities of daily living; BMI = body mass index; CI = confidence interval; CRP = C-reactive protein; FEV_1_ = forced expiratory volume in 1s; HDL = high-density lipoprotein; MMSE = Mini-Mental State Examination. ^a^Including BMI but not obesity. ^b^All risk factors included in model, except BMI. ^c^Corrected for height, measured at fourth clinic (2002–2004). Missing risk factor data imputed using multiple imputation with 20 imputations. Impaired physical functioning—at least one ADL. Impaired cognitive functioning—MMSE < 27. Some participants have both impaired physical and cognitive functioning. *p* Values = pairwise tests for heterogeneity using Cochran’s *Q* test.

In mutually adjusted analysis, physical inactivity, hypertension, and low FEV_1_ at age 50 years remained risk factors for both outcomes at follow-up, although the association of physical inactivity with cognitive impairment was borderline (Figure 1). This association was significant when adjusted for other health behaviors (as well as demographics) but not for biomedical factors (OR 1.29 95% CI 1.02–1.63; Supplementary Table 4). Compared with minimally adjusted analysis, fewer modifiable risk factors at age 50 years were associated with impaired physical functioning in the mutually adjusted models. We found heterogeneity for the depression and BMI coefficients with impaired physical, but not with impaired cognitive, functioning associated with depression and higher BMI (ORs respectively 1.72 95% CI 1.46–2.03 and 1.29 95% CI 1.16–1.44) in the mutually adjusted analysis (Supplementary Tables 2 and 3).

Hypertension was associated with an increase in the prevalence of both impaired physical and cognitive functioning of about 5% from 14% in those without hypertension whereas sufficiently active, compared with inactive, participants had more than 10% lower prevalence of impaired physical and cognitive functioning (Supplementary Table 1). In addition, the 20% of participants with the lowest FEV values had a prevalence of impaired physical and cognitive functioning of 21% and 23% respectively, compared with the other 80% of participants who had a prevalence of impaired physical and cognitive functioning of about 13%.

Further results are tabulated in the Supplementary Material. Supplementary Table 4 shows the associations of modifiable risk factors with impaired physical and cognitive functioning with alternative adjustment to avoid potential collider and mediator effects. In addition to adjustment for demographic factors, the effects of health behaviors are mutually adjusted, and likewise, the effects of biomedical factors are mutually adjusted. Supplementary Tables 5–8 show sensitivity analyses using the LOCF method instead of multiple imputation. For impaired physical functioning (Supplementary Table 7), low fruit and vegetable consumption (OR 1.26 95% CI 1.04–1.52) and obesity (OR 1.79 95% CI 1.34–2.38) were risk factors in the mutually adjusted model, but hypertension was not (OR 1.07, 95% CI 0.86–1.33). For impaired cognitive functioning (Supplementary Table 8) physical inactivity (OR 1.24 95% CI 0.94–1.63) was not a risk factor, and obesity (OR 0.66 95% CI 0.45–0.96) was protective in mutually adjusted analysis.

## Discussion

We examined prospective associations between modifiable risk factors at age 50 years and age-related functional loss 18 years later: impaired physical functioning and impaired cognitive functioning. We found that physical inactivity, hypertension, and low FEV_1_ at age 50 years were each associated prospectively with both functioning outcomes after adjustment for the other candidate risk factors (for the physical inactivity–impaired cognitive functioning association, adjusted *p* = .055). Depression and obesity at age 50 years were associated with risk of impaired physical functioning but not with impaired cognitive functioning. On the basis that our observations are causal with respect to physical and cognitive decline, physical inactivity, hypertension, and poor lung function around age 50 years appear to be important targets for prevention.

To our knowledge, this is the first study to compare modifiable risk factor effects in both major functional domains, physical and cognitive (4). The novel evidence adds weight to the view that physical and cognitive functional declines can both potentially be prevented or delayed by midlife approaches to risk factor reduction (19). Our findings are observational and do not demonstrate reversibility. However, intervention trials to date have recruited older participants beyond the midlife stage, have not come within reach of the 18-year period of follow-up in the present cohort study, and have focused on cognition decline rather than on functional decline more broadly (20). Our finding that hypertension was a robust risk factor for physical as well as cognitive impairments thus extends the trial evidence in respect of blood pressure reduction (2).

Observational data show that physical activity is protective for dementia (3,21). We add to the evidence that inactivity in midlife is also linked to increased risk for impaired physical functioning in later life. Our finding for impaired cognitive functioning was clear in the minimally adjusted analysis (OR 1.54 95% CI 1.23–1.93), remained significant in a model adjusted for demographics and health behaviors (Supplementary Table 4) but not after further adjustment for biomedical factors including some which may mediate the benefits of physical activity into conserved cognition (OR 1.28, 95% CI 0.99–1.65; Supplementary Table 3). Lung function and cognitive function have been shown to be correlated across the life course (22). Our prospective study presents a coherent picture, with physical inactivity and poor lung function each associated with future impairments in the physical and cognitive domains. Smoking at age 50 was associated with later impairments of physical functioning after minimal adjustment but not after further adjustment for other factors including FEV_1_. The present null finding in respect of smoking and cognition could be due to competing risks (23,24).

We found the expected association of obesity with impairment in physical functioning, but no link with cognitive functioning (25). Current evidence is mixed: A recent large study challenges the conventional view with findings that high BMI is a protective factor for cognitive impairment (26). Mixed findings were also obtained for depressive symptoms, which were associated with later impairment of physical functioning, but not as anticipated with cognitive functioning (27). Many but not all of such prospective studies (28) involve participants considerably older at baseline than in the present study. There was a modest association between CRP level and future impaired physical functioning, significant in the minimally but not mutually adjusted model. A cross-sectional association between CRP and physical performance measures has been observed, but the level did not predict change in performance in that study (29).

Key strengths of this study are the substantial risk factor exposure data collected in midlife together with long follow-up. The baseline of aging studies may be considerably later in the life course, and therefore Whitehall II is valuable for studies of exposure effects before or early in the processes leading to functional loss. Although the cohort is not representative of the general population in terms of risk factor distributions and health status, the associations of vascular risk factors are similar to those seen in community-based cohort studies and the effects we estimate in the present study are likely to be broadly generalizable (30). Notably, we found that the three risk factors that emerge as important determinants of age-related functioning were weakly correlated with one another (all Pearson correlation coefficients <.15).

There was a low prevalence of impaired cognitive functioning in our relatively young sample. Many studies use an MMSE score of less than 24 (31,32) to define impaired cognition. Here the score distribution led us to utilize the MMSE score less than 27 cut point in order to increase sensitivity (12,33) (Supplementary Figure 2). Participants who satisfied our MMSE criterion were generally not severely cognitively impaired, and this may have contributed to type 2 errors (false positives) in estimates including the null relation with depressive symptoms and borderline association with physical activity (27). The relative youthfulness of the study sample has the advantage that dementia-related loss to follow-up is small.

To reduce potential bias in estimates of coefficients and their standard errors, we imputed missing values. We included many predictive auxiliary variables to reduce bias (18). To assess the validity of multiple imputation for handling missing data, we compared results based on imputation with those using the LOCF method as a sensitivity analysis. A few of the associations observed using multiple imputation differed in the sensitivity analysis. In part, discrepancies probably arose because the LOCF method assumes risk factor levels stay constant with increasing age. In this context, multiple imputation is the preferred approach for handling missing data. As expected, there was excess mortality among those lost to follow-up after age 50 (*n* = 702, hazard ratio 2.37 95% CI 1.9–3.0, adjusted for age, sex, and ethnicity). However, the group was only 10% of the target sample, and selection bias in the estimated associations is likely to be small. This study examines 12 risk factor effects based on pre-existing hypotheses. We checked for multiple testing and confirmed the reported associations except for one mutually adjusted association, between hypertension and cognitive impairment, which became marginally nonsignificant (34).

Delaying or preventing age-related functional impairments is a health policy research priority (19). Here we add to the sparse evidence that the same modifiable risk factors impact upon impaired physical and cognitive functioning in the same setting. Whether the interventions take place in clinical or public health settings, or in both, this study suggests that blood pressure control, adequate physical activity, and maintenance of lung function should be at the centre of a unified prevention policy. It remains unclear how effective such policies could be. An early estimate attributed up to half the prevalence of Alzheimer’s disease to seven modifiable risk factors (diabetes, midlife hypertension, midlife obesity, physical inactivity, depression, smoking, and low educational attainment), whereas more recent calculations are more cautious (21,35). In the present study, there were important excesses in crude prevalence of impaired physical and cognitive functioning, as defined, according to hypertension (5% excess), lung function below the first quintile (9% excess), and physical inactivity (about 12% excess) versus about 14% among the comparison groups.

In conclusion, our observational findings support the proposition that modifiable risk factor reduction in midlife may have benefit for maintaining global functioning in older people. We found support for blood pressure control, adequate physical activity, and maintenance of lung function as key targets for delaying onset of impaired physical and cognitive functioning. Replication of our findings in other cohorts is needed, however, to support an evidence-based, unified approach to functional loss in older people.

## Supplementary Material

Supplementary material can be found at: http://biomedgerontology.oxfordjournals.org/

## Funding

This work was supported by the British Heart Foundation (RG/13/2/30098), British Medical Research Council (K013351), the British Health and Safety Executive, the British Department of Health, the British Stroke Association (TSA 2008/05), the US National Heart, Lung, and Blood Institute (R01HL036310), and the US National Institute on Aging (R01AG013196 and R01AG034454). M.K. is additionally supported by a professorial fellowship from the Economic and Social Research Council and by NordForsk.

## Supplementary Material

Supplementary Data
