# Supplementary material for: Midlife Risk Factors for Impaired Physical and Cognitive Functioning at Older Ages: A Cohort Study
Source: J Gerontol A Biol Sci Med Sci. 2016 Jun 6;72(2):237–42. doi: 10.1093/gerona/glw092 (PMC5233910; doi:10.1093/gerona/glw092)
Supplement: Supplementary Data [file supp_72_2_237__index.html]

Midlife Risk Factors for Impaired Physical and Cognitive Functioning at Older Ages: A Cohort Study — Midlife Risk Factors for Impaired Physical and Cognitive Functioning at Older Ages: A Cohort Study — Supplementary Data 

# Midlife Risk Factors for Impaired Physical and Cognitive Functioning at Older Ages: A Cohort Study

## Supplementary Data

Data files

- Supplementary Data - Supplementary Data
