# Supplementary material for: Midlife Risk Factors for Impaired Physical and Cognitive Functioning at Older Ages: A Cohort Study
Source: J Gerontol A Biol Sci Med Sci. 2016 Jun 6;72(2):237–42. doi: 10.1093/gerona/glw092 (PMC5233910; doi:10.1093/gerona/glw092)
Supplement: Supplementary Data [file supp_glw092_Risk_factors_impairment_paper___Supplementary_data_FINAL.docx]

**Supplementary Material**

Midlife risk factors for impaired physical and cognitive functioning at older ages: a cohort study

Eric J. Brunner, Catherine A. Welch, Martin J. Shipley, Sara Ahmadi-Abhari, Archana Singh-Manoux, Mika Kivimäki

Research Department of Epidemiology and Public Health, University College London, UK

**Supplementary Figure 1: Risk factors, functional outcome measures and covariates according to domains of the International Classification of Functioning, Disability and Health model**

Health condition:
Myocardial infarction or stroke at age 50 years
Depression at age 50 years

Personal factors:
Age
Sex
Ethnicity
Marital status
Highest academic qualification
Civil service grade at age 50 years

Fruit and vegetable consumption at age 50 years
Smoking status at age 50 years
Alcohol consumption at age 50 years
Sleep duration at age 50 years
Physical activity at age 50 years

Body function & structure:
**Body function**
Mini mental state examination at ages 55-84

Body mass index at age 50 years
Obese at age 50 years
Systolic blood pressure at age 50 years
Total:high density lipoprotein cholesterol ratio at age 50 years
Fasting glucose at age 50 years
Forced expiratory volume in 1 second at age 50 years
C-reactive protein at age 50 years

**Body structure**No factors specified

Participation:
No factors specified

Environmental factors
No factors specified

Activity:
Activities of daily living at ages 55-84

**Supplementary Figure 2: Distribution of participants according to Activities of Daily Living impairments (A) and Mini Mental State Examination score (B) at time of outcome measurement (2007-2009 or 2012-2013) N=6,316**

**A.**

**B.**

**Supplementary Table 1: Prevalence of impaired physical and cognitive functioning at time of outcome measurement (2007-2009 or 2012-2013) by modifiable participant characteristics - observed data before imputation (with row percentages).**

|  | | | **Total  (N=6,316)** | **Impaired physical functioning (N=980)** | | **Impaired cognitive functioning (N=927)** | |
| --- | --- | --- | --- | --- | --- | --- | --- |
|  | |  |  | **n (%)** | **p-value^a^** | **n (%)** | **p-value^a^** |
| **Health behaviors** | | |  |  |  |  |  |
| Physical activity | | Sufficiently active | 2,837 | 411 (14.5) | <0.001 | 407 (14.3) | <0.001 |
|  |  | Moderately active | 853 | 166 (19.5) |  | 146 (17.1) |  |
|  |  | Inactive | 564 | 137 (24.3) |  | 160 (28.4) |  |
|  |  | *Missing* | *2,062* | *266 (12.9)* |  | *214 (10.4)* |  |
|  | |  |  |  |  |  |  |
| Fruit and vegetable consumption | | Daily | 2,953 | 467 (15.8) | 0.013 | 470 (15.9) | 0.005 |
|  |  | Less | 1,564 | 293 (18.7) |  | 301 (19.2) |  |
|  |  | *Missing* | *1,799* | *220 (12.2)* |  | *156 (8.7)* |  |
|  | |  |  |  |  |  |  |
| Smoker status | | Never smoker | 2,289 | 359 (15.7) | <0.001 | 407 (17.8) | 0.012 |
|  |  | Ex-smoker | 1,676 | 278 (16.6) |  | 246 (14.7) |  |
|  |  | Current smoker | 508 | 117 (23.0) |  | 97 (19.1) |  |
|  |  | *Missing* | *1,843* | *226 (12.3)* |  | *177 (9.6)* |  |
|  | |  |  |  |  |  |  |
| Alcohol consumption | | None | 607 | 112 (18.5) | 0.268 | 144 (23.7) | <0.001 |
|  |  | Moderate | 2,716 | 433 (15.9) |  | 442 (16.3) |  |
|  |  | Heavy | 733 | 127 (17.3) |  | 104 (14.2) |  |
|  |  | *Missing* | *2,260* | *308 (13.6)* |  | *237 (10.5)* |  |
|  | |  |  |  |  |  |  |
| Average hours sleep on a week night | | Normal | 1,903 | 303 (15.9) | 0.056 | 332 (17.4) | 0.662 |
|  |  | Abnormal | 1,263 | 234 (18.5) |  | 228 (18.1) |  |
|  |  | *Missing* | 3,150 | *443 (14.1)* |  | *367 (11.7)* |  |
|  |  | |  |  |  |  |  |
| **Biomedical factors** | | |  |  |  |  |  |
| Stroke or myocardial infarction | | Yes | 63 | 12 (19.0) | 0.437 | 13 (20.6) | 0.179 |
|  |  | No | 6,253 | 968 (15.5) |  | 914 (14.6) |  |
|  | |  |  |  |  |  |  |
| Depression | | Yes | 1,368 | 300 (21.9) | <0.001 | 207 (15.1) | 0.520 |
|  |  | No | 3,711 | 510 (13.7) |  | 589 (15.9) |  |
|  |  | *Missing* | *1,237* | *170 (13.7)* |  | *131 (10.6)* |  |
|  | |  |  |  |  |  |  |
| BMI (kg m^-2^) | | Normal (BMI<25) | 1,976 | 264 (13.4) | <0.001 | 331 (16.8) | 0.591 |
|  |  | Overweight (25≤BMI<30) | 1,492 | 277 (18.6) |  | 268 (18.0) |  |
|  |  | Obese (BMI≥30) | 369 | 116 (31.4) |  | 67 (18.2) |  |
|  |  | *Missing* | *2,479* | *323 (13.0)* |  | *261 (10.5)* |  |
|  | |  |  |  |  |  |  |
| Hypertension | | Yes | 999 | 193 (19.3) | <0.001 | 188 (18.8) | <0.001 |
|  |  | No | 4,828 | 707 (14.6) |  | 660 (13.7) |  |
|  |  | *Missing* | *489* | *80 (16.4)* |  | *79 (16.2)* |  |
|  | |  |  |  |  |  |  |
| Total:HDL cholesterol ratio | | M>5.9/ F>4.7^b^ | 546 | 95 (17.4) | 0.050 | 80 (14.7) | 0.444 |
|  |  | M≤5.9/ F≤4.7 | 2,195 | 309 (14.1) |  | 294 (13.4) |  |
|  |  | *Missing* | *3,575* | *576 (16.1)* |  | *553 (15.5)* |  |
|  | |  |  |  |  |  |  |
| Fasting glucose  (mmol l^-1^) | | M>5.6/ F>5.3^b^ | 441 | 66 (15.0) | 0.087 | 37 (8.4) | 0.073 |
|  |  | M≤5.6/ F≤5.3 | 1,790 | 214 (12) |  | 203 (11.3) |  |
|  |  | *Missing* | *4,085* | *700 (17.1)* |  | *687 (16.8)* |  |
|  | |  |  |  |  |  |  |
| FEV_1_^c^ (l) | | M<2.8/ F<1.8^b^ | 873 | 182 (20.8) | <0.001 | 200 (22.9) | <0.001 |
|  |  | M≥2.8/ F≥1.8 | 3,491 | 468 (13.4) |  | 429 (12.3) |  |
|  |  | *Missing* | *1,952* | *330 (16.9)* |  | *298 (15.3)* |  |
|  | |  |  |  |  |  |  |
| CRP (mg l^-1^) | | M>2.1/ F>3.2^b^ | 432 | 83 (19.2) | <0.001 | 42 (9.7) | 0.459 |
|  |  | M≤2.1/ F≤3.2 | 1,744 | 188 (10.8) |  | 191 (11.0) |  |
|  |  | *Missing* | *4,140* | *709 (17.1)* |  | *694 (16.8)* |  |

^a^ Χ^2^ test for heterogeneity of prevalence in each exposure group
^b^ Cut-off used to identify highest 20% of measurements for male (M) and female (F) participants, lowest 20% for FEV_1_
^c^ Corrected for height, measured at the fourth clinic (2002-2004)
BMI: body mass index; HDL: high-density lipoprotein; FEV_1_: forced expiratory volume in 1 second; CRP: C-reactive protein

Impaired physical functioning – at least one activity of daily living. Impaired cognitive functioning – mini mental state examination score<27. Some participants have both impaired physical and cognitive functioning.

**Supplementary Table 2: Logistic regression analysis of the association between risk factors recorded at age 50 years and impaired physical functioning at time of outcome measurement (2007-2009 or 2012-2013). N=6,316**

|  | | **Minimally adjusted** | | **Mutually adjusted** | |
| --- | --- | --- | --- | --- | --- |
|  |  | **OR (95% CI)** | **p-value^a^** | **OR (95% CI)** | **p-value^a^** |
| **Demographic factors** | |  |  |  |  |
| Time from age 50^b^ to outcome measurement (years) | | 0.99 (0.98,1.00) | 0.219 | 0.96 (0.94,0.98) | <0.001 |
|  |  |  |  |  |  |
| Sex | Male | Ref | <0.001 | Ref | <0.001 |
|  | Female | 1.31 (1.13,1.52) |  | 0.62 (0.48,0.81) |  |
|  |  |  |  |  |  |
| Ethnicity | White | Ref | 0.821 | Ref | 0.041 |
|  | South Asian | 1.11 (0.81,1.51) |  | 0.71 (0.49,1.03) |  |
|  | Black | 1.11 (0.74,1.68) |  | 0.58 (0.36,0.93) |  |
|  | Other | 0.79 (0.33,1.88) |  | 0.61 (0.25,1.51) |  |
|  |  |  |  |  |  |
| Marital status | Married/ cohabiting | Ref | <0.001 | Ref | 0.009 |
|  | Single | 1.37 (1.11,1.68) |  | 1.30 (1.04,1.62) |  |
|  | Divorced/ widowed | 1.52 (1.21,1.92) |  | 1.37 (1.07,1.76) |  |
|  |  |  |  |  |  |
| Highest ever academic qualification | Degree | Ref | <0.001 | Ref | 0.403 |
|  | School | 1.25 (1.07,1.45) |  | 1.08 (0.90,1.29) |  |
|  | None | 1.73 (1.33,2.25) |  | 1.24 (0.90,1.72) |  |
|  |  |  |  |  |  |
| Last known grade | 1-2 (High) | Ref | <0.001 | Ref | 0.205 |
|  | 3-5 | 1.23 (1.06,1.44) |  | 1.05 (0.87,1.26) |  |
|  | 6 (Low) | 1.77 (1.40,2.24) |  | 1.30 (0.96,1.75) |  |
|  |  |  |  |  |  |
| **Health behaviors** | |  |  |  |  |
| Physical activity | Sufficiently active | Ref | <0.001 | Ref | 0.001 |
|  | Moderately active | 1.40 (1.14,1.72) |  | 1.29 (1.04,1.60) |  |
|  | Inactive | 1.85 (1.49,2.30) |  | 1.50 (1.19,1.90) |  |
|  |  |  |  |  |  |
| Fruit and vegetable consumption | Daily | Ref | 0.005 | Ref | 0.344 |
|  | Less | 1.27 (1.08,1.49) |  | 1.09 (0.91,1.31) |  |
|  |  |  |  |  |  |
| Smoking status | Never smoker | Ref | <0.001 | Ref | 0.159 |
|  | Ex-smoker | 1.10 (0.92,1.31) |  | 1.00 (0.82,1.22) |  |
|  | Current smoker | 1.59 (1.27,1.99) |  | 1.27 (0.98,1.65) |  |
|  |  |  |  |  |  |
| Alcohol consumption | None | Ref | 0.684 | Ref | 0.758 |
|  | Moderate | 0.94 (0.74,1.20) |  | 1.10 (0.85,1.43) |  |
|  | Heavy | 1.03 (0.76,1.40) |  | 1.10 (0.79,1.53) |  |
|  |  |  |  |  |  |
| Average number of hours sleep on a week night | Normal | Ref | 0.022 | Ref | 0.591 |
|  | Abnormal | 1.23 (1.03,1.47) |  | 1.05 (0.87,1.27) |  |
|  | |  |  |  |  |
| **Biomedical factors** | |  |  |  |  |
| Stroke or myocardial infarction | | 1.32 (0.70,2.49) | 0.388 | 0.98 (0.50,1.91) | 0.954 |
|  | |  |  |  |  |
| Depression | | 1.72 (1.47,2.01) | <0.001 | 1.72 (1.46,2.03) | <0.001 |
|  | |  |  |  |  |
| BMI (per 1SD kg m^-2^ increase) | | 1.45 (1.34,1.56) | <0.001 | 1.29 (1.16,1.44) | <0.001 |
|  | |  |  |  |  |
| Obese^c^ (BMI≥30kg m^-2^) | | 2.25 (1.80,2.83) | <0.001 | 1.58 (1.20,2.08) | 0.001 |
|  | |  |  |  |  |
| Hypertension | | 2.22 (1.75,2.80) | <0.001 | 1.80 (1.39,2.33) | <0.001 |
|  | |  |  |  |  |
| Log total:HDL cholesterol ratio (per 1SD increase) | | 1.15 (1.04,1.27) | 0.009 | 0.95 (0.85,1.07) | 0.393 |
|  | |  |  |  |  |
| Log fasting glucose (per 1SD increase) | | 1.19 (1.05,1.35) | 0.006 | 1.10 (0.97,1.25) | 0.133 |
|  | |  |  |  |  |
| FEV_1_^d^ (per 1l decrease) | | 1.73 (1.49,2.00) | <0.001 | 1.51 (1.28,1.78) | <0.001 |
|  | |  |  |  |  |
| Log C-reactive protein (per 1SD increase) | | 1.36 (1.18,1.58) | <0.001 | 1.12 (0.94,1.34) | 0.204 |

^a^ Wald test (joint Wald test for risk factors with multiple categories) for overall association between risk factors and outcome
^b^ Data collection closest to age 50
^c^ All risk factors included in mutually adjusted model, except BMI
^d^ Corrected for height, measured at the fourth clinic (2002-2004)

BMI: body mass index; HDL: high-density lipoprotein; FEV_1_: forced expiratory volume in 1 second; OR: odds ratio; CI: confidence interval; SD: standard deviation
Missing risk factor data imputed using multiple imputation with 20 imputations.
Minimal adjustment adjusts for age, sex and ethnicity. Mutual adjustment adjusts for all risk factors, including BMI but not obesity status.
Impaired physical functioning- at least one activity of daily living, present in 980 participants.

**Supplementary Table 3: Logistic regression analysis of the association between risk factors recorded at age 50 years and impaired cognitive functioning at time of outcome measurement (2007-2009 or 2012-2013). N=6,316**

|  | | **Minimally adjusted** | | **Mutually adjusted** | |
| --- | --- | --- | --- | --- | --- |
|  |  | **OR (95% CI)** | **p-value^a^** | **OR (95% CI)** | **p-value^a^** |
| **Demographic factors** | |  |  |  |  |
| Time from age 50^b^ to outcome measurement (years) | | 1.06 (1.04,1.07) | <0.001 | 1.03 (1,1.05) | 0.030 |
|  |  |  |  |  |  |
| Sex | Male | Ref | 0.023 | Ref | <0.001 |
|  | Female | 1.20 (1.02,1.40) |  | 0.45 (0.34,0.61) |  |
|  |  |  |  |  |  |
| Ethnicity | White | Ref | <0.001 | Ref | <0.001 |
|  | South Asian | 5.60 (4.38,7.16) |  | 3.73 (2.71,5.15) |  |
|  | Black | 7.90 (5.70,10.97) |  | 4.10 (2.80,6.00) |  |
|  | Other | 2.22 (1.11,4.42) |  | 1.84 (0.88,3.86) |  |
|  |  |  |  |  |  |
| Marital status | Married/ cohabiting | Ref | 0.361 | Ref | 0.753 |
|  | Single | 1.17 (0.93,1.46) |  | 1.03 (0.81,1.31) |  |
|  | Divorced/ widowed | 1.09 (0.84,1.42) |  | 0.91 (0.68,1.20) |  |
|  |  |  |  |  |  |
| Highest ever academic qualification | Degree | Ref | <0.001 | Ref | 0.009 |
|  | School | 1.46 (1.24,1.73) |  | 1.03 (0.85,1.24) |  |
|  | None | 3.19 (2.47,4.13) |  | 1.51 (1.12,2.05) |  |
|  |  |  |  |  |  |
| Last known grade | 1-2 (High) | Ref | <0.001 | Ref | <0.001 |
|  | 3-5 | 1.90 (1.59,2.28) |  | 1.83 (1.49,2.24) |  |
|  | 6 (Low) | 5.85 (4.58,7.47) |  | 5.21 (3.86,7.04) |  |
|  | |  |  |  |  |
| **Health behaviors** | |  |  |  |  |
| Physical activity | Sufficiently active | Ref | <0.001 | Ref | 0.136 |
|  | Moderately active | 1.03 (0.83,1.27) |  | 1.01 (0.81,1.25) |  |
|  | Inactive | 1.52 (1.21,1.91) |  | 1.27 (0.99,1.62) |  |
|  |  |  |  |  |  |
| Fruit and vegetable consumption | Daily | Ref | 0.128 | Ref | 0.954 |
|  | Less | 1.14 (0.96,1.35) |  | 1.01 (0.84,1.20) |  |
|  |  |  |  |  |  |
| Smoking status | Never smoker | Ref | 0.265 | Ref | 0.444 |
|  | Ex-smoker | 0.93 (0.78,1.12) |  | 0.89 (0.73,1.08) |  |
|  | Current smoker | 1.19 (0.91,1.57) |  | 0.90 (0.66,1.22) |  |
|  |  |  |  |  |  |
| Alcohol consumption | None | Ref | 0.377 | Ref | 0.266 |
|  | Moderate | 0.84 (0.66,1.08) |  | 1.14 (0.87,1.49) |  |
|  | Heavy | 0.84 (0.62,1.13) |  | 1.32 (0.94,1.83) |  |
|  |  |  |  |  |  |
| Average number of hours sleep on a week night | Normal | Ref | 0.737 | Ref | 0.617 |
|  | Abnormal | 1.03 (0.86,1.25) |  | 0.95 (0.78,1.16) |  |
|  | |  |  |  |  |
| **Biomedical factors** | |  |  |  |  |
| Stroke or myocardial infarction | | 1.64 (0.85,3.15) | 0.137 | 1.40 (0.71,2.79) | 0.334 |
|  | |  |  |  |  |
| Depression | | 0.95 (0.79,1.13) | 0.534 | 1.02 (0.85,1.22) | 0.859 |
|  | |  |  |  |  |
| BMI (per 1SD kg m^-2^ increase) | | 1.01 (0.93,1.11) | 0.746 | 1.02 (0.91,1.16) | 0.689 |
|  | |  |  |  |  |
| Obese^c^ (BMI≥30kg m^-2^) | | 1.02 (0.77,1.34) | 0.888 | 0.98 (0.72,1.35) | 0.921 |
|  | |  |  |  |  |
| Hypertension | | 1.57 (1.11,2.21) | 0.012 | 1.57 (1.07,2.31) | 0.023 |
|  | |  |  |  |  |
| Log total:HDL cholesterol ratio (per 1SD increase) | | 0.98 (0.88,1.09) | 0.710 | 0.96 (0.85,1.09) | 0.539 |
|  | |  |  |  |  |
| Log fasting glucose (per 1SD increase) | | 0.90 (0.77,1.06) | 0.208 | 0.89 (0.75,1.06) | 0.173 |
|  | |  |  |  |  |
| FEV_1_^d^ (per 1l decrease) | | 1.44 (1.20,1.71) | <0.001 | 1.31 (1.08,1.59) | 0.006 |
|  | |  |  |  |  |
| Log C-reactive protein (per 1SD increase) | | 0.94 (0.81,1.10) | 0.448 | 0.86 (0.72,1.04) | 0.116 |

^a^ Wald test (joint Wald test for risk factors with multiple categories) for overall association between risk factors and outcome
^b^ Data collection closest to age 50
^c^ All risk factors included in mutually adjusted model, except BMI
^d^ Corrected for height measured at the fourth clinic (2002-2004)

BMI: body mass index; HDL: high-density lipoprotein; FEV_1_: forced expiratory volume in 1 second; OR: odds ratio; CI: confidence interval; SD: standard deviation.

Missing risk factor data imputed using multiple imputation with 20 imputations.
Minimal adjustment adjusts for age, sex and ethnicity. Mutual adjustment adjusts for all risk factors, including BMI but not obesity status.
Impaired cognitive functioning- mini mental state examination score <27, present in 927 participants..

**Supplementary Figure 3: Prospective associations of modifiable risk factors at age 50 years with impaired physical and cognitive functioning in 2007-2009 or 2012-2013. N=6,316, using one minimally adjusted^a^ model for each risk factor.**

**
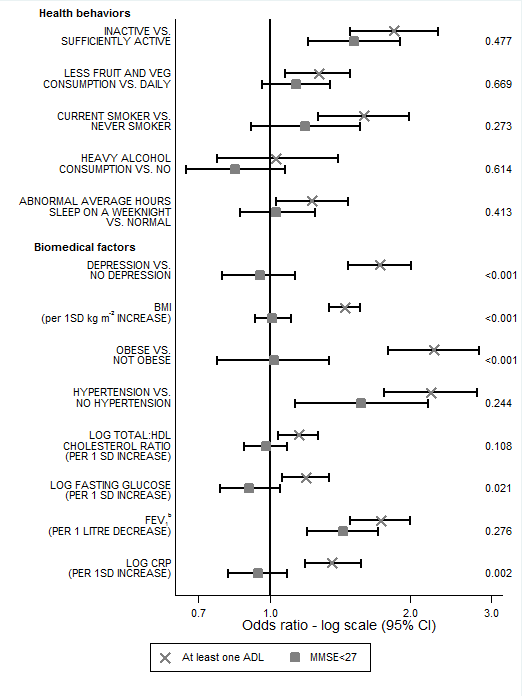
**

Footnote

ADL: activities of daily living; MMSE: mini mental state examination

P-values: pairwise tests for heterogeneity using Cochran's Q test

BMI: body mass index; HDL: high-density lipoprotein; FEV_1_: forced expiratory volume in 1 second; CRP: C-reactive protein; CI: confidence interval

^a^ Minimally adjusted odds ratios are adjusted for age, sex and ethnicity

^b^ Corrected for height, measured at fourth clinic (2002-2004)

Missing risk factor data imputed using multiple imputation with 20 imputations.
Impaired physical functioning – at least one ADL. Impaired cognitive functioning – MMSE<27. Some participants have both impaired physical and cognitive functioning.

**Supplementary Table 4: Logistic regression analysis of the association between, a) health behaviours (adjusted for demographic factors and other health behaviours only) and, b) biomedical factors (adjusted for demographic factors and other biomedical factors only) recorded at age 50 years and impaired physical functioning and impaired cognitive functioning at time of outcome measurement (2007-2009 or 2012-2013). N=6,316**

|  | | **Impaired physical functioning (N=980)** | | **Impaired cognitive functioning (N=927)** | |
| --- | --- | --- | --- | --- | --- |
|  |  | **OR (95% CI)** | **p-value^a^** | **OR (95% CI)** | **p-value^a^** |
| **Health behaviors** | |  |  |  |  |
| Physical activity | Sufficiently active | Ref | <0.001 | Ref | 0.084 |
|  | Moderately active | 1.37 (1.12, 1.69) |  | 1.00 (0.81, 1.24) |  |
|  | Inactive | 1.71 (1.37, 2.13) |  | 1.29 (1.02, 1.63) |  |
|  |  |  |  |  |  |
| Fruit and vegetable consumption | Daily | Ref | 0.168 | Ref | 0.895 |
|  | Less | 1.12 (0.95, 1.33) |  | 0.99 (0.83, 1.18) |  |
|  |  |  |  |  |  |
| Smoking status | Never smoker | Ref | 0.038 | Ref | 0.275 |
|  | Ex-smoker | 1.06 (0.88, 1.28) |  | 0.86 (0.72, 1.04) |  |
|  | Current smoker | 1.36 (1.07, 1.72) |  | 0.89 (0.66, 1.19) |  |
|  |  |  |  |  |  |
| Alcohol consumption | None | Ref | 0.708 | Ref | 0.447 |
|  | Moderate | 1.05 (0.81, 1.36) |  | 1.09 (0.84, 1.41) |  |
|  | Heavy | 1.14 (0.82, 1.58) |  | 1.23 (0.89, 1.70) |  |
|  |  |  |  |  |  |
| Average number of hours sleep on a week night | Normal | Ref | 0.067 | Ref | 0.775 |
|  | Abnormal | 1.18 (0.99, 1.41) |  | 0.97 (0.80, 1.18) |  |
|  | |  |  |  |  |
| **Biomedical factors** | |  |  |  |  |
| Stroke or myocardial infarction | | 0.95 (0.49, 1.86) | 0.882 | 1.40 (0.70, 2.79) | 0.339 |
|  | |  |  |  |  |
| Depression | | 1.77 (1.51, 2.08) | <0.001 | 1.02 (0.85, 1.22) | 0.846 |
|  | |  |  |  |  |
| BMI (per 1SD kg m^-2^ increase) | | 1.29 (1.16, 1.44) | <0.001 | 1.03 (0.91, 1.16) | 0.646 |
|  | |  |  |  |  |
| Obese^c^ (BMI≥30kg m^-2^) | | 1.59 (1.21, 2.11) | 0.001 | 0.99 (0.72, 1.35) | 0.947 |
|  | |  |  |  |  |
| Hypertension | | 1.78 (1.37, 2.31) | <0.001 | 1.57 (1.07, 2.30) | 0.022 |
|  | |  |  |  |  |
| Log total:HDL cholesterol ratio (per 1SD increase) | | 0.96 (0.86, 1.08) | 0.536 | 0.95 (0.84, 1.08) | 0.448 |
|  | |  |  |  |  |
| Log fasting glucose (per 1SD increase) | | 1.10 (0.97, 1.25) | 0.138 | 0.90 (0.76, 1.06) | 0.195 |
|  | |  |  |  |  |
| FEV_1_^d^ (per 1l decrease) | | 1.55 (1.32,1.83) | <0.001 | 1.31 (1.09,1.59) | 0.005 |
|  | |  |  |  |  |
| Log C-reactive protein (per 1SD increase) | | 1.14 (0.95, 1.36) | 0.159 | 0.87 (0.72, 1.04) | 0.112 |

^a^ Wald test (joint Wald test for risk factors with multiple categories) for overall association between risk factors and outcome
^b^ Data collection closest to age 50
^c^ All risk factors included in mutually adjusted model, except BMI
^d^ Corrected for height measured at the fourth clinic (2002-2004)

BMI: body mass index; HDL: high-density liproprotein; FEV_1_: forced expiratory volume in 1 second; OR: odds ratio; CI: confidence interval; SD: standard deviation
Missing risk factor data imputed using multiple imputation with 20 imputations.
Impaired physical functioning – at least one activity of daily living. Impaired cognitive functioning – mini mental state evaluation<27. Some participants have both impaired physical and cognitive functioning

**Sensitivity analysis**

In Tables 5 to 8, missing values have been replaced with observed values from the closest recorded value before age 50 (last observation carried forward method) or if missing, the closest recorded value after age 50, when available.

**Supplementary Table 5: Prevalence of impaired physical and cognitive functioning at time of outcome measurement (2007-2009 or 2012-2013) by non-modifiable participant characteristics (with row percentages) N=6,316**

|  | | **Total  (N= 6,316)** | **Impaired physical functioning (N=980)** | | **Impaired cognitive functioning (N=927)** | |
| --- | --- | --- | --- | --- | --- | --- |
|  |  |  | **n (%)** | **p-value^a^** | **n (%)** | **p-value^a^** |
| Age group (years) at time of outcome measurement^b^ | 55-64 | 1,795/1,802 | 277 (15.4) | <0.001 | 175 (9.7) | <0.001 |
|  | 65-69 | 1,834/1,855 | 231 (12.6) |  | 213 (11.5) |  |
|  | 70-74 | 1,375/1,390 | 242 (17.6) |  | 271 (19.5) |  |
|  | 75-79 | 1,092/1,042 | 194 (17.8) |  | 229 (22.0) |  |
|  | 80 + | 220/227 | 36 (16.4) |  | 39 (17.2) |  |
|  |  |  |  |  |  |  |
| Sex | Male | 4,516 | 653 (14.5) | <0.001 | 603 (13.4) | <0.001 |
|  | Female | 1,800 | 327 (18.2) |  | 324 (18.0) |  |
|  |  |  |  |  |  |  |
| Ethnicity^c^ | White | 5,819 | 895 (15.4) | 0.648 | 703 (12.1) | <0.001 |
|  | South Asian | 292 | 50 (17.1) |  | 129 (44.2) |  |
|  | Black | 160 | 29 (18.1) |  | 84 (52.5) |  |
|  |  |  |  |  |  |  |
| Marital status at age 50 | Married/ cohabiting | 4,951 | 707 (14.3) | <0.001 | 710 (14.3) | 0.210 |
|  | Single | 815 | 155 (19.0) |  | 123 (15.1) |  |
|  | Divorced/ widowed | 550 | 118 (21.5) |  | 94 (17.1) |  |
|  |  |  |  |  |  |  |
| Highest ever academic qualification | Degree | 2,520 | 336 (13.3) | <0.001 | 280 (11.1) | <0.001 |
|  | School | 3,052 | 494 (16.2) |  | 462 (15.1) |  |
|  | None | 463 | 100 (21.6) |  | 130 (28.1) |  |
|  | *Missing* | *281* | *50 (17.8)* |  | *55 (19.6)* |  |
|  |  |  |  |  |  |  |
| Last known grade | 1-2 (High) | 2,717 | 359 (13.2) | <0.001 | 217 (8.0) | <0.001 |
|  | 3-5 | 2,798 | 447 (16.0) |  | 418 (14.9) |  |
|  | 6 (Low) | *801* | 174 (21.7) |  | *292 (36.5)* |  |

^a^ Χ^2^ test for heterogeneity of prevalence in each exposure group
^b^ The figures in the age group total column are impaired physical functioning total/impaired cognitive functioning total
^c^ 45 participants with ‘other’ ethnicity
Missing values have been replaced with the closest recorded value before age 50 (or if missing, after age 50)
Impaired physical functioning – at least one activity of daily living. Impaired cognitive functioning – mini mental state evaluation<27. Some participants have both impaired physical and cognitive functioning

**Supplementary Table 6: Prevalence of impaired physical and cognitive functioning at time of outcome measurement (2007-2009 or 2012-2013) by modifiable participant characteristics (with row percentages). N=6,316**

|  | | **Total  (N=6,316)** | **Impaired physical functioning (N=980)** | | **Impaired cognitive functioning (N=927)** | |
| --- | --- | --- | --- | --- | --- | --- |
|  |  |  | **n, (%)** | **p-value^a^** | **n (%)** | **p-value^a^** |
| **Health behaviors** | |  |  |  |  |  |
| Physical activity | Sufficiently active | 4,139 | 572 (13.8) | <0.001 | 524 (12.7) | <0.001 |
|  | Moderately active | 1,323 | 223 (16.9) |  | 193 (14.6) |  |
|  | Inactive | 854 | 185 (21.7) |  | 210 (24.6) |  |
|  |  |  |  |  |  |  |
| Fruit and vegetable consumption | Daily | 4,091 | 593 (14.5) | 0.002 | 556 (13.6) | 0.001 |
|  | Less | 2,225 | 387 (17.4) |  | 371 (16.7) |  |
|  |  |  |  |  |  |  |
| Smoker status | Never smoker | 3,257 | 462 (14.2) | <0.001 | 508 (15.6) | 0.001 |
|  | Ex-smoker | 2,336 | 360 (15.4) |  | 293 (12.5) |  |
|  | Current smoker | 722 | 157 (21.7) |  | 125 (17.3) |  |
|  | *Missing* | *1* | *1 (100.0)* |  | *1 (100.0)* |  |
|  |  |  |  |  |  |  |
| Alcohol consumption | None | 954 | 158 (16.6) | 0.360 | 208 (21.8) | <0.001 |
|  | Moderate | 4,197 | 632 (15.1) |  | 582 (13.9) |  |
|  | Heavy | 1,164 | 190 (16.3) |  | 136 (11.7) |  |
|  | *Missing* | *1* | *0 (0.0)* |  | *1 (100.0)* |  |
|  |  |  |  |  |  |  |
| Average hours sleep on a week night | Normal | 3,915 | 554 (14.2) | <0.001 | 554 (14.2) | 0.128 |
|  | Abnormal | 2,399 | 424 (17.7) |  | 373 (15.5) |  |
|  | *Missing* | *2* | *2 (100.0)* |  | *0 (0.0)* |  |
|  |  |  |  |  |  |  |
| **Biomedical factors** | |  |  |  |  |  |
| Stroke or myocardial infarction | Yes | 63 | 12 (19.0) | 0.437 | 13 (20.6) | 0.179 |
|  | No | 6,253 | 968 (15.5) |  | 914 (14.6) |  |
|  |  |  |  |  |  |  |
| Depression | Yes | 1,698 | 366 (21.6) | <0.001 | 251 (14.8) | 0.886 |
|  | No | 4,618 | 614 (13.3) |  | 676 (14.6) |  |
|  |  |  |  |  |  |  |
| BMI (kg m^-2^) | Normal (BMI<25) | 3,330 | 410 (12.3) | <0.001 | 454 (13.6) | 0.044 |
|  | Overweight (25≤BMI<30) | 2,397 | 406 (16.9) |  | 382 (15.9) |  |
|  | Obese (BMI≥30) | 588 | 164 (27.9) |  | 91 (15.5) |  |
|  | *Missing* | *1* | *0 (0.0)* |  | *0 (0.0)* |  |
|  |  |  |  |  |  |  |
| Hypertension | Yes | 1,416 | 271 (19.1) | <0.001 | 264 (18.6) | <0.001 |
|  | No | 4,900 | 709 (14.5) |  | 663 (13.5) |  |
|  |  |  |  |  |  |  |
| Total:HDL cholesterol ratio | M>5.9/ F>4.7^b^ | 1,245 | 224 (18.0) | 0.006 | 184 (14.8) | 0.819 |
|  | M≤5.9/ F≤4.7 | 4,985 | 740 (14.8) |  | 724 (14.5) |  |
|  | *Missing* | *86* | *16 (18.6)* |  | *19 (22.1)* |  |
|  |  |  |  |  |  |  |
| Fasting glucose  (mmol l^-1^) | M>5.3/ F>5.4^b^ | 1,145 | 196 (17.1) | 0.090 | 185 (16.2) | 0.078 |
|  | M≤5.3/ F≤5.4 | 5,062 | 765 (15.1) |  | 715 (14.1) |  |
|  | *Missing* | *109* | *19 (17.4)* |  | *27 (24.8)* |  |
|  |  |  |  |  |  |  |
| FEV_1_^c^ (l) | M<2.8/ F<1.8^b^ | 873 | 182 (20.8) | <0.001 | 200 (22.9) | <0.001 |
|  | M≥2.8/ F≥1.8 | 3,491 | 468 (13.4) |  | 429 (12.3) |  |
|  | *Missing* | *1,952* | *330 (16.9)* |  | *298 (15.3)* |  |
|  |  |  |  |  |  |  |
| CRP (mg l^-1^) | M>2.1/ F>3.2^b^ | 1,231 | 256 (20.8) | <0.001 | 185 (15.0) | 0.566 |
|  | M≤2.1/ F≤3.2 | 4,950 | 696 (14.1) |  | 712 (14.4) |  |
|  | *Missing* | *135* | *28 (20.7)* |  | *30 (22.2)* |  |

^a^ Χ^2^ test for heterogeneity of prevalence in each exposure group
^b^ Cut-off used to identify highest 20% of measurements for male (M) and female (F) participants, lowest 20% for FEV_1_
^c^ Corrected for height, measured at the fourth clinic (2002-2004)
BMI: body mass index; HDL: high-density lipoprotein; FEV_1_: forced expiratory volume in 1 second; CRP: C-reactive protein
Missing values have been replaced with the closest recorded value before age 50 (or if missing, after age 50)
Impaired physical functioning – at least one activity of daily living. Impaired cognitive functioning – mini mental state evaluation<27. Some participants have both impaired physical and cognitive functioning

**Supplementary Table 7: Logistic regression analysis of the association between risk factors recorded at age 50 years and impaired physical functioning at the time of outcome measurement (2007-2009 or 2012-2013). Sample size for minimally adjusted analysis shown with each risk factor. For mutually adjusted analysis, N=4,167**

|  | | **Minimally adjusted** | | **Mutually adjusted** | |
| --- | --- | --- | --- | --- | --- |
|  |  | **OR (95% CI)** | **p-value^a^** | **OR (95% CI)** | **p-value^a^** |
| **Demographic factors** | |  |  |  |  |
| Time from age 50^b^ to outcome measurement (years) (N=6,316) | | 0.99 (0.98,1.00) | 0.218 | 0.98 (0.96,1.00) | 0.032 |
|  |  |  |  |  |  |
| Sex (N=6,316) | Male | Ref | <0.001 | Ref | 0.002 |
|  | Female | 1.31 (1.13,1.52) |  | 0.66 (0.50,0.88) |  |
|  |  |  |  |  |  |
| Ethnicity (N=6,316) | White | Ref | 0.812 | Ref | 0.251 |
|  | South Asian | 1.11 (0.81,1.52) |  | 0.78 (0.49,1.26) |  |
|  | Black | 1.11 (0.74,1.68) |  | 0.77 (0.43,1.38) |  |
|  | Other | 0.80 (0.34,1.89) |  | 0.19 (0.03,1.43) |  |
|  |  |  |  |  |  |
| Marital status (N=6,316) | Married/ cohabiting | Ref | <0.001 | Ref | 0.020 |
|  | Single | 1.36 (1.12,1.65) |  | 1.26 (0.98,1.63) |  |
|  | Divorced/widowed | 1.55 (1.24,1.94) |  | 1.44 (1.08,1.93) |  |
|  |  |  |  |  |  |
| Highest ever academic qualification (N=6,035) | Degree | Ref | <0.001 | Ref | 0.782 |
|  | School | 1.24 (1.07,1.44) |  | 1.08 (0.87,1.33) |  |
|  | None | 1.73 (1.33,2.24) |  | 1.04 (0.70,1.53) |  |
|  |  |  |  |  |  |
| Last known grade (N=6,316) | 1-2 (High) | Ref | <0.001 | Ref | 0.663 |
|  | 3-5 | 1.23 (1.06,1.44) |  | 0.98 (0.78,1.22) |  |
|  | 6 (Low) | 1.77 (1.40,2.24) |  | 1.13 (0.78,1.61) |  |
|  |  |  |  |  |  |
| **Health behaviors** | |  |  |  |  |
| Physical activity (N=6,316) | Sufficiently active | Ref | <0.001 | Ref | 0.055 |
|  | Moderately active | 1.28 (1.07,1.52) |  | 1.19 (0.95,1.49) |  |
|  | Inactive | 1.68 (1.38,2.04) |  | 1.34 (1.03,1.75) |  |
|  |  |  |  |  |  |
| Fruit and vegetable consumption (N=6,316) | Daily | Ref | 0.001 | Ref | 0.016 |
|  | Less | 1.28 (1.11,1.47) |  | 1.26 (1.04,1.52) |  |
|  |  |  |  |  |  |
| Smoking status (N=6,315) | Never smoker | Ref | <0.001 | Ref | 0.349 |
|  | Ex-smoker | 1.14 (0.98,1.33) |  | 1.04 (0.85,1.27) |  |
|  | Current smoker | 1.69 (1.38,2.07) |  | 1.25 (0.92,1.70) |  |
|  |  |  |  |  |  |
| Alcohol consumption (N=6,315) | None | Ref | 0.439 | Ref | 0.424 |
|  | Moderate | 0.96 (0.79,1.17) |  | 1.16 (0.88,1.52) |  |
|  | Heavy | 1.08 (0.85,1.37) |  | 1.03 (0.74,1.44) |  |
|  |  |  |  |  |  |
| Average number of hours sleep on a week night (N=6,314) | Normal | Ref | <0.001 | Ref | 0.123 |
|  | Abnormal | 1.30 (1.13,1.49) |  | 1.15 (0.96,1.38) |  |
|  |  |  |  |  |  |
| **Biomedical factors** | |  |  |  |  |
| Stroke or myocardial infarction (N=6,316) | | 1.32 (0.70,2.49) | 0.389 | 1.33 (0.52,3.38) | 0.547 |
|  | |  |  |  |  |
| Depression (N=6,316) | | 1.76 (1.53,2.04) | <0.001 | 1.52 (1.26,1.84) | <0.001 |
|  | |  |  |  |  |
| BMI (per 1SD kg m^-2^ increase) (N=6,315) | | 1.38 (1.30,1.47) | <0.001 | 1.31 (1.19,1.44) | <0.001 |
|  | |  |  |  |  |
| Obese^c^ (BMI≥30kg m^-2^) (N=6,315) | | 2.24 (1.84,2.74) | <0.001 | 1.79 (1.34,2.38) | <0.001 |
|  | |  |  |  |  |
| Hypertension (N=6,316) | | 1.42 (1.21,1.65) | <0.001 | 1.07 (0.86,1.33) | 0.543 |
|  | |  |  |  |  |
| Log total:HDL cholesterol ratio (per 1SD increase) (N=6,230) | | 1.19 (1.11,1.28) | <0.001 | 0.98 (0.89,1.08) | 0.700 |
|  | |  |  |  |  |
| Log fasting glucose (per 1SD increase) (N=6,207) | | 1.14 (1.07,1.21) | <0.001 | 1.06 (0.97,1.15) | 0.185 |
|  | |  |  |  |  |
| FEV_1_^d^ (per 1l decrease) (N=4,364) | | 1.71 (1.47,1.99) | <0.001 | 1.50 (1.27,1.76) | <0.001 |
|  | |  |  |  |  |
| Log C-reactive protein (per 1SD increase) (N=6,181) | | 1.29 (1.20,1.38) | <0.001 | 1.05 (0.95,1.16) | 0.363 |

^a^ Wald test (joint Wald test for risk factors with multiple categories) for overall association between risk factors and outcome
^b^ Data collection closest to age 50

^c^ All risk factors included in mutually adjusted model, except BMI
^d^ Corrected for height, measured at the fourth clinic (2002-2004)

BMI: body mass index; HDL: high-density lipoprotein; FEV_1_: forced expiratory volume in 1 second; OR: odds ratio; CI: confidence interval; SD: standard deviation.

Missing values have been replaced with the closest recorded value before age 50 (or if missing, after age 50)
Minimally adjusted for age, sex and ethnicity. Mutually adjusted for all risk factors, except obesity.
Impaired physical functioning – at least one activity of daily living, present in 980 participants among the full sample of 6316 persons.

**Supplementary Table 8: Logistic regression analysis of the association between risk factors recorded at age 50 years and impaired cognitive functioning at time of outcome measurement (2007-2009 or 2012-2013. Sample size for minimally adjusted analysis shown with each risk factor. For mutually adjusted analysis, N=4,167**

|  | | **Minimally adjusted** | | **Mutually adjusted** | |
| --- | --- | --- | --- | --- | --- |
|  |  | **OR (95% CI)** | **p-value^a^** | **OR (95% CI)** | **p-value^a^** |
| **Demographic factors** | |  |  |  |  |
| Time from age 50^b^ to outcome measurement (years) (N=6,316) | | 1.06 (1.04,1.07) | <0.001 | 1.05 (1.03,1.07) | <0.001 |
|  |  |  |  |  |  |
| Sex (N=6,316) | Male | Ref | 0.023 | Ref | <0.001 |
|  | Female | 1.20 (1.03,1.40) |  | 0.47 (0.35,0.64) |  |
|  |  |  |  |  |  |
| Ethnicity (N=6,316) | White | Ref | <0.001 | Ref | <0.001 |
|  | South Asian | 5.63 (4.40,7.21) |  | 4.09 (2.79,5.99) |  |
|  | Black | 7.92 (5.71,10.99) |  | 4.28 (2.63,6.98) |  |
|  | Other | 2.21 (1.11,4.41) |  | 2.90 (1.24,6.81) |  |
|  |  |  |  |  |  |
| Marital status (N=6,316) | Married/ cohabiting | Ref | 0.331 | Ref | 0.305 |
|  | Single | 1.18 (0.95,1.47) |  | 1.09 (0.83,1.43) |  |
|  | Divorced/ widowed | 1.06 (0.82,1.36) |  | 0.80 (0.57,1.12) |  |
|  |  |  |  |  |  |
| Highest ever academic qualification (N=6,035) | Degree | Ref | <0.001 | Ref | 0.005 |
|  | School | 1.46 (1.23,1.72) |  | 1.17 (0.93,1.48) |  |
|  | None | 3.21 (2.47,4.17) |  | 1.82 (1.26,2.62) |  |
|  |  |  |  |  |  |
| Last known grade (N=6,316) | 1-2 (High) | Ref | <0.001 | Ref | <0.001 |
|  | 3-5 | 1.90 (1.59,2.28) |  | 1.78 (1.38,2.28) |  |
|  | 6 (Low) | 5.85 (4.58,7.47) |  | 4.56 (3.19,6.54) |  |
|  |  |  |  |  |  |
| **Health behaviors** | |  |  |  |  |
| Physical activity (N=6,316) | Sufficiently active | Ref | <0.001 | Ref | 0.291 |
|  | Moderately active | 0.98 (0.81,1.18) |  | 1.09 (0.87,1.38) |  |
|  | Inactive | 1.54 (1.26,1.89) |  | 1.24 (0.94,1.63) |  |
|  |  |  |  |  |  |
| Fruit and vegetable consumption (N=6,316) | Daily | Ref | 0.102 | Ref | 0.709 |
|  | Less | 1.13 (0.98,1.32) |  | 1.04 (0.85,1.27) |  |
|  |  |  |  |  |  |
| Smoking status (N=6,315) | Never smoker | Ref | 0.038 | Ref | 0.705 |
|  | Ex-smoker | 0.92 (0.78,1.08) |  | 0.94 (0.76,1.16) |  |
|  | Current smoker | 1.25 (1.00,1.56) |  | 1.07 (0.79,1.45) |  |
|  |  |  |  |  |  |
| Alcohol consumption (N=6,315) | Abstain | Ref | 0.108 | Ref | 0.934 |
|  | Moderate | 0.81 (0.67,0.99) |  | 0.96 (0.74,1.24) |  |
|  | Heavy | 0.81 (0.63,1.05) |  | 0.99 (0.70,1.40) |  |
|  |  |  |  |  |  |
| Average number of hours sleep on a week night (N=6,314) | Normal | Ref | 0.112 | Ref | 0.238 |
|  | Abnormal | 1.13 (0.97,1.31) |  | 1.12 (0.93,1.36) |  |
|  |  |  |  |  |  |
| **Biomedical factors** | |  |  |  |  |
| Stroke or myocardial infarction (N=6,316) | | 1.64 (0.85,3.14) | 0.137 | 1.09 (0.35,3.35) | 0.880 |
|  | |  |  |  |  |
| Depression (N=6,316) | | 1.03 (0.88,1.22) | 0.685 | 1.03 (0.83,1.28) | 0.791 |
|  | |  |  |  |  |
| BMI (per 1SD kg m^-2^ increase (N=6,315) | | 1.04 (0.97,1.12) | 0.305 | 0.98 (0.88,1.10) | 0.744 |
|  | |  |  |  |  |
| Obese^c^ (BMI≥30kg m^-2^) (N=6,315) | | 1.02 (0.79,1.32) | 0.870 | 0.66 (0.45,0.96) | 0.032 |
|  | |  |  |  |  |
| Hypertension (N=6,316) | | 1.30 (1.10,1.53) | 0.002 | 1.36 (1.08,1.70) | 0.008 |
|  | |  |  |  |  |
| Log total:HDL cholesterol ratio (per 1SD increase) (N=6,230) | | 0.97 (0.90,1.05) | 0.502 | 0.93 (0.84,1.04) | 0.206 |
|  | |  |  |  |  |
| Log fasting glucose (per 1SD increase) (N=6,207) | | 1.06 (0.99,1.13) | 0.123 | 1.02 (0.93,1.12) | 0.614 |
|  | |  |  |  |  |
| FEV_1_^d^ (per 1l decrease) (N=4,364) | | 1.48 (1.26,1.75) | <0.001 | 1.29 (1.08,1.54) | 0.005 |
|  | |  |  |  |  |
| Log C-reactive protein (per 1SD increase) (N=6,181) | | 1.04 (0.96,1.12) | 0.348 | 1.04 (0.94,1.16) | 0.448 |

^a^ Wald test (joint Wald test for risk factors with multiple categories) for overall association between risk factors and outcome
^b^ Data collection closest to age 50
^c^ All risk factors included in mutually adjusted model, except BMI
^d^ Corrected for height, measured at the fourth clinic (2002-2004)

BMI: body mass index; FEV_1_: forced expiratory volume; OR: odds ratio; CI: confidence interval; SD: standard deviation
Missing values have been replaced with the closest recorded value before age 50 (or if missing, after age 50)
Minimally adjusted for age, sex and ethnicity. Mutually adjusted for all risk factors except obesity.
Impaired cognitive functioning- mini mental state examination score <27, present in 927 participants among the full sample of 6316 persons.
